# Supplementary material for: Challenges experienced by health care providers working in both hospital and home-based palliative care units in Dhaka city: A multi-center based cross-sectional study
Source: PLoS One. 2024 Sep 26;19(9):e0306790. doi: 10.1371/journal.pone.0306790 (PMC11426436; doi:10.1371/journal.pone.0306790)
Supplement: S1 Table — (DOCX) [file pone.0306790.s001.docx]

Distribution of respondents as per mostly handled patients (n=160)

| Name of diagnosis | Frequency (f) | Percentages (%) |
| --- | --- | --- |
| Late-stage Cancer | 159 | 99.4 |
| End stage CVD | 45 | 28.1 |
| Stroke & CNSD | 58 | 36.3 |
| End stage CKD | 57 | 35.6 |
| Geriatric | 95 | 59.4 |
| CP | 39 | 24.4 |

Distribution of respondents as per most predominant symptom seen in patients (n=160)

| Symptoms | Frequency (f) | Percentages (%) |
| --- | --- | --- |
| Pain | 153 | 95.6 |
| Fatigue | 77 | 48.1 |
| Breathlessness | 100 | 62.5 |
| Disorientation | 82 | 51.3 |
| Constipation | 87 | 54.4 |
| Hemorrhage | 50 | 31.3 |
| Wound | 54 | 33.8 |
| Vomiting | 43 | 26.9 |
