# Supplementary material for: Challenges experienced by health care providers working in both hospital and home-based palliative care units in Dhaka city: A multi-center based cross-sectional study
Source: PLoS One. 2024 Sep 26;19(9):e0306790. doi: 10.1371/journal.pone.0306790 (PMC11426436; doi:10.1371/journal.pone.0306790)
Supplement: S2 Table — (DOCX) [file pone.0306790.s002.docx]

**Table : Relationship between professional level and professional satisfaction**

|  | | | |
| --- | --- | --- | --- |
|  | B | SE | Sig |
| Hospital type | 0.773 | 0.050 | 0.000 (Anova) |
|  | 0.813 | 0.109 | 0.000 (coefi) |
| Working experience | 1.074 | 0.76 | 0.000 (coefi) |
|  | 0.304 | 0.045 |  |
| Income | 1.018 | 0.065 | 0.000 (coefi) |
|  | 0.488 | 0.065 |  |
